# Cognitive reserve and network efficiency as compensatory mechanisms of the effect of aging on phonemic fluency

**DOI:** 10.18632/aging.202177

**Published:** 2020-11-17

**Authors:** Lissett Gonzalez-Burgos, José Barroso, Daniel Ferreira

**Affiliations:** 1 Department of Clinical Psychology, Psychobiology and Methodology, Faculty of Health Science, Section of Psychology and Speech Therapy, University of La Laguna, La Laguna, Tenerife, Spain; 2 Division of Clinical Geriatrics, Center for Alzheimer Research, Department of Neurobiology, Care Sciences, and Society, Karolinska Institutet, Stockholm, Sweden

**Keywords:** compensation, phonemic fluency, aging, random forest, graph theory

## Abstract

Compensation in cognitive aging is a topic of recent interest. However, factors contributing to cognitive compensation in functions such as phonemic fluency (PF) are not completely understood. Using cross-sectional data, we investigated cognitive reserve (CR) and network efficiency in young (32-58 years) versus old (59-84 years) individuals with high versus low performance in PF. ANCOVA was used to investigate the interaction between CR, age, and performance in PF. Random forest and graph theory analyses were conducted to study the contribution of cognition to PF and efficiency measures, respectively. Higher CR increased performance in PF and reduced age-related differences in PF. A slightly higher number of cognitive functions contributed to performance in high CR groups. The networks were more integrated in high CR individuals, both in the older age and high-performance groups. The strength and segregation of the networks were decreased in high-performance groups with high CR. We conclude that PF decreases less with age in individuals with higher CR, possibly due to a greater capacity to recruit non-linguistic cognitive networks, and efficient use of language networks, thereby integrating information in a rapid way across less fragmented networks. High CR and network efficiency seem to be important factors for cognitive compensation.

## INTRODUCTION

Language is essential for human communication. Although many cognitive functions decline with age, language is one of the few functions that can resist the onslaught of aging [1, 2]. An explanation for this is that language abilities are broadly distributed through different neural networks across the brain [3]. Comprehension, semantic abilities, and vocabulary remain rather stable or even improve with age [2, 4]. In contrast, verbal fluency and naming decline with age [5].

It has been suggested that brain functional reorganization is the mechanism through which cognitive performance is maintained with increasing age [6]. Compensation refers to the maintenance or enhancement of performance by recruiting brain areas or networks not normally used for a specific task, as a response to brain deterioration [7] or high cognitive demands [8]. From a cognitive perspective, compensation can be approached by investigating how different cognitive functions are associated with or contribute to language abilities [9]. In particular, performance in phonemic fluency has been associated with processing speed [10–12], attention [13, 14], lexical access [15], executive functions [14, 16–19], and memory [14, 20]. Due to the complexity of human cognition, an interesting approach is to investigate the contribution of different cognitive functions to verbal fluency by using multivariate methods for data analysis. We previously used the random forest multivariate method to investigate the contribution of 45 cognitive variables to phonemic fluency [9]. In younger individuals, lexical access, working memory, processing speed, and visuoconstructive abilities were the most contributing functions to performance in phonemic fluency. In older individuals, the same functions contributed to phonemic fluency but, interestingly, cognitive functions such as premotor and visuospatial abilities contributed to phonemic fluency as well. In that previous study, compensation was suggested as the mechanism possibly underlying the findings. However, further research is needed to elucidate the factors involved in these compensatory mechanisms.

Previous studies have linked compensatory mechanisms to the concepts of cognitive reserve (CR) and neural efficiency. CR is “the adaptability of cognitive processes that helps to explain differential susceptibility of cognitive abilities or day-to-day function to brain aging, pathology, or insult” [7]. People with higher CR produce more words in phonemic fluency [21–25]. Furthermore, people with higher CR have greater neural efficiency [26, 27]. Graph theory is a popular approach to compute and analyze different measures of efficiency. For instance, the measures of average strength, average global efficiency, and transitivity are commonly used to investigate the magnitude of the associations, network integration, and network segregation, respectively. Integration is the capacity of the brain to rapidly combine information from distributed brain regions [28]. Segregation is the biologically meaningful feature of the brain to enable highly specialized processing through densely interconnected communities of regions [29]. There are numerous studies investigating efficiency on neuroimaging data, both in normal aging [30] and neurodegenerative disorders [31, 32]. However, to our knowledge, only two studies investigated efficiency on cognitive data, and these investigated individuals with epilepsy and did not focus on compensatory mechanisms [33, 34]. Applying graph theory analysis on cognitive data may be useful to characterize compensatory mechanisms associated with cognitive reserve, which is indeed a cognitive construct.

In the current study, we sought to advance our understanding of factors contributing to cognitive compensation. The overall goal was to investigate how CR and efficiency levels contribute to phonemic fluency differently in people with high versus low fluency performance and in younger versus older individuals. Firstly, we investigated the effects of CR, performance level, and age on phonemic fluency. Secondly, we studied the contribution of other linguistic and non-linguistic cognitive functions to phonemic fluency. Thirdly, we compared efficiency measures of average strength, global efficiency, and transitivity in individuals with high and low performance in phonemic fluency. We hypothesized that older adults would perform worse than younger adults in verbal fluency, but this difference would be minimized by high CR levels and high efficiency of cognitive networks. In other words, high CR levels and network efficiency would help to maintain high performance in older adults, thus contributing to compensate for the negative effect of age.

## RESULTS

**Figure 1:**
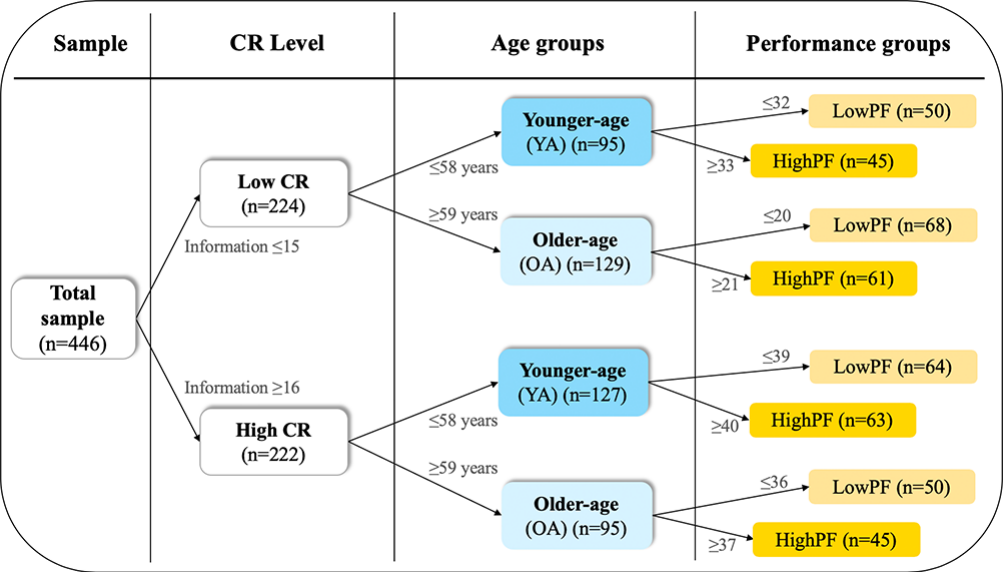
**Cohort stratification.** The cohort was stratified into groups of CR, performance in phonemic fluency, and age, using the median values for these variables as shown next to the arrows in the Figure. CR, cognitive reserve. PF, phonemic fluency performance.

To address the three aims of this study, we stratified the cohort into groups of CR, performance in phonemic fluency, and age as detailed in Figure 1. Table 1 shows the demographic characteristics and Supplementary Table 1 shows cognitive performance across the CR, performance, and age groups.

**Table 1:** Demographic characteristics and performance in phonemic fluency by study group.

			**Low Cognitive Reserve (lowCR)**				**High Cognitive Reserve (highCR)**				
			**Younger-age (YA, n=95)**		**Older-age (OA, n=129)**		**Younger-age (YA, n=127)**		**Older-age (OA, n=95)**		
			**Low performance (lowPF)**	**High performance (highPF)**	**Low performance (lowPF)**	**High performance (highPF)**	**Low performance (lowPF)**	**High performance (highPF)**	**Low performance (lowPF)**	**High performance (highPF)**	
			**M(SD)/count(%)**	**M(SD)/count(%)**	**M(SD)/count(%)**	**M(SD)/count(%)**	**M(SD)/count(%)**	**M(SD)/count(%)**	**M(SD)/count(%)**	**M(SD)/count(%)**	***p*-value**
*n*			50	45	68	61	64	63	50	45	
Age, years (min-max)			46.9 (5.7) (37-58) ^b,c,f,g^	46.6 (5.7) (34-58) ^b,c,f,g^	68.8 (4.8) (59-79) ^d,e^	69.3 (4.6) (60-80) ^d,e,g^	48.0 (5.7) (38-58) ^f,g^	48.5 (6.0) (32-58) ^f,g^	67.8 (5.3) (59-79)	66.1 (6.0) (59-84)	p<0.001
Sex (female, count (%))			39 (78%) ^d-g^	31 (69%) ^d,f^	42 (62%) ^d^	41 (67%) ^d,f^	22 (34%)	30 (48%)	19 (38%)	21 (47%)	p<0.001
Education level											p<0.001
Illiteracy			0	0	5	2	0	0	0	0	
Unfinished primary studies			1	2	27	20	0	0	3	0	
Completed primary studies			38	26	28	29	14	8	12	7	
Completed secondary studies			8	12	7	8	26	18	15	7	
University studies			3	5	1	2	24	37	20	30	
WAIS-III Information			10.1 (3.1) ^d-g^	11.3 (2.8) ^b,d-g^	8.8 (2.8) ^d-g^	9.7 (3.2) ^d-g^	20.4 (2.8)	21.4 (3.1)	19.8 (2.8)	20.9 (3.0)	p<0.001
MMSE			28,7 (1,2) ^b^	28,9 (1,4) ^b,c^	27,1 (1,6) ^c-e^	27,9 (1,4)	29,2 (0,9)	29,3 (0,9)	28,5 (1,5)	28,7 (1,1)	p<0.001
(min-max)			25 - 30	25 - 30	24 - 30	25 - 30	27 - 30	27 - 30	25 - 30	25 - 30	
Phonemic fluency			23.9 (4.9) ^a-g^	38.9 (6.2) ^b-g^	14.2 (4.2) ^c-g^	27.6 (5.2) ^d,e,g^	32.0 (5.3) ^e-g^	48.8 (8.3) ^f^	27.8 (6.7) ^g^	47.1 (8.4)	p<0.001
(min-max)			(14-32)	(33-67)	(5-20)	(21-43)	(16-39)	(40-71)	(12-36)	(37-74)	

MMSE: Mini-Mental State Examination; WAIS-III: Wechsler Adult Intelligence Scale; Third edition. ^a^ Significantly different from YA+highPF+lowCR, ^b^ Significantly different from OA+lowPF+lowCR, ^c^ Significantly different from OA+highPF+lowCR, ^d^ Significantly different from YA+lowPF+highCR, ^e^ Significantly different from YA+highPF+highCR, ^f^ Significantly different from OA+lowPF+highCR, ^g^ Significantly different from OA+highPF+highCR.

**Figure 2:**
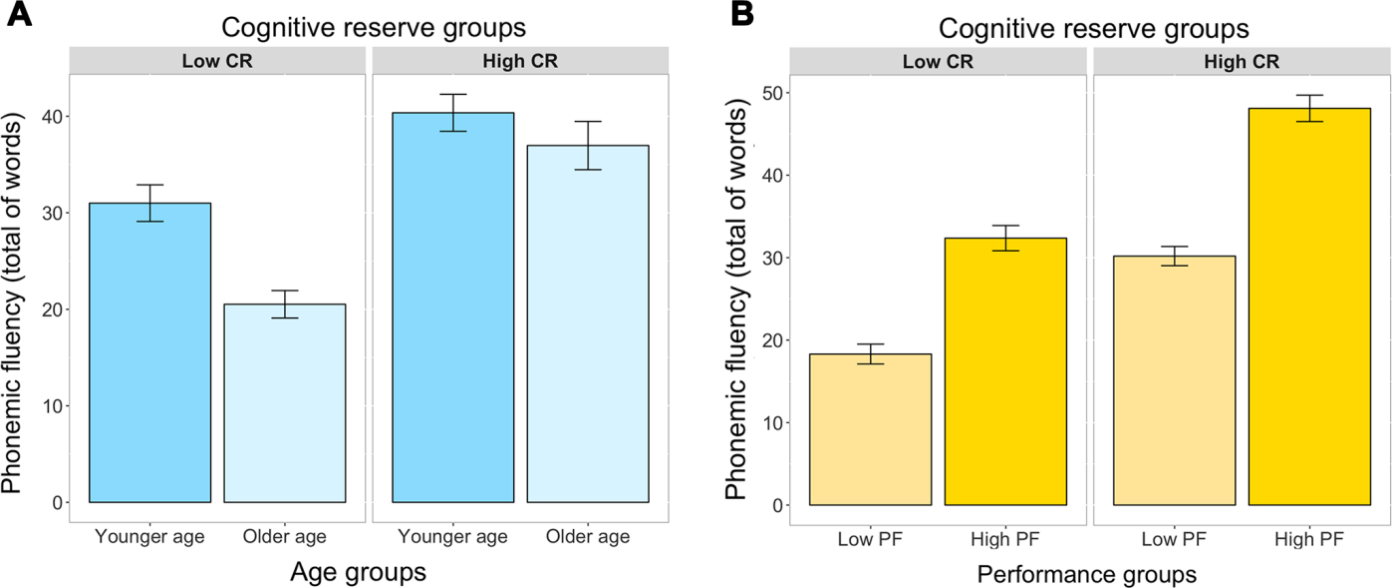
Interaction between CR levels and age (**A**), and between CR levels and performance groups (**B**), in the prediction of phonemic fluency (ANCOVA). Bars represent the mean of words produced and the jack-knifes represent the 95% confidence intervals. Panel **A** represents the interaction between CR and age. Panel **B** represents the interaction between CR and performance groups. CR, cognitive reserve; YA, younger age; OA, older age; Low PF, low phonemic fluency performance; High PF, high phonemic fluency performance.

Regarding our first aim, the ANCOVA did not show any significant triple interaction among CR, performance, and age groups (*p*=0.084). However, the ANCOVA showed a significant interaction between CR and age groups (F_(3, 442)_=38.68; *p*<0.001) (Figure 2A), and between CR and performance groups (F_(3, 442)_=10.34; *p*<0.01) (Figure 2B). We elaborate on these two interactions in the next two sections, respectively.

### High cognitive reserve reduces age-related differences in phonemic fluency

The significant interaction between CR and age revealed that the younger age (YA) group outperformed the older age (OA) group (p<0.001), but this difference was smaller in the high CR (highCR) group than in the low CR (lowCR) group (Figure 2A). Hence, higher CR reduces age-related differences.

To answer our second aim, four random forest regression models were performed separately within each group (YA+lowCR, OA+lowCR, YA+highCR, and OA+highCR) (Table 2A). For a description of the cognitive variables (predictors) included in the random forests and their abbreviation please see Table 3. In the OA+highCR group, the model explained 38% of the variance and 24 variables contributed to performance in phonemic fluency. The most important variables in predicting performance were Stroop (Colors and Inhibition) and BNT (Table 2A). In the YA+highCR group, the model explained 19% of the variance and 23 variables contributed to performance. The most important variables in predicting performance were Stroop (Colors) and Digit span backward. In the OA+lowCR, the model explained 19% of the variance and 18 variables contributed to performance. The most important variables in predicting performance were CTT-Part 1 and Stroop (Colors). In the YA+lowCR group, the model explained 24% of the variance and 22 variables contributed to performance. The most important variables in predicting performance were Stroop (Words) and Digit span backward. Hence, a slightly higher number of variables contribute to performance in the highCR groups, and the strength of this contribution is the greatest in OA+highCR individuals (as reflected by the % of variance). When entering sex as an extra predictor, these results were virtually the same (data not shown), demonstrating that sex does not have any confounding effect in these models.

**Table 2:** Contribution of cognitive variables to phonemic fluency (random forest regression models).

	**A) CR by age groups.**					**B) CR by performance groups.**			
	**Low CR (n=224)**		**High CR (n=222)**			**Low CR (n=224)**		**High CR (n=222)**	
	**YA**	**OA**	**YA**	**OA**		**LowPF**	**HighPF**	**LowPF**	**HighPF**
**Group size**	95	129	127	95		118	106	114	108
**Explained variance**	24%	19%	19%	38%		50%	45%	17%	13%
**Predictors**									
BNT	8	15	5	25		20	14	13	
PCV - Decision time				7			6		
PCV - Motor time	6					1	16	17	
PASAT			4						4
STROOP Words	26	9	13	25		13	31	17	14
STROOP Colors	7	27	26	37		29	19	10	
STROOP Inhibition	10	9	13	31		13	15	4	31
TMT A	11	10	6	12		12	23	17	17
CTT - Part 1		31	4	9		35	24		
CTT - Part 2	6	20				28	23		
FRT				4		13		1	3
JLOT - First half		1				5		18	4
JLOT - Second half		6							
Digit Span forward	6	4	8	14			4		
Digit Span backward	20		22	4		1		4	1
Spatial Span forward	2		8				10		6
Spatial Span backward			5			4	4		
LM A - Immediate	2						4		
LM B1 - Immediate	2		7	11		16	4		
LM B2 - Immediate			7	9			10		
LM A - Delay							2		
LM B - Delay			5	12			7		2
LM A - Recognition	8	2					1		
LM B - Recognition			9	4			4	4	
TAVEC 1^st^ trial			4	7					
TAVEC Learning				18		2			
TAVEC Short delay				5		6		4	
TAVEC Short delay-Clues								6	
TAVEC Long delay				4		5	13		
TAVEC Long delay-Clues				1		3	4	9	
TAVEC Intrusions Delay	3								
TAVEC Intrusions Delay-Clues	1	7	2			2	4	4	
TAVEC Perseverations	9		8				2		
TAVEC Recog. Correct	2								
TAVEC Recog. False Positive		9		6		2			
VR I – Total score		5	6			14	6		22
VR II – Total score		12		4		22	11		11
VR-Copying	2			4		3	13	3	
VR Total Recog.		7	1	5		7	4	8	
VR False Positive		3				7		22	
VR Visual discrimination	6	2	3				2		
Luria’s HAM Right	2		13			11	12		4
Luria’s HAM Left	2		2	3		21	19	9	4
Luria’s - Coordination						25	29		
Block Design WAIS	4					10	24	8	
Total of variables contributing to the prediction of PF	**22**	**18**	**23**	**24**		**28**	**32**	**19**	**13**
*Importance*	Not important		<10	10 - 19			20 - 29	>30	

Panel A) Cognitive reserve by age groups. Panel B) Cognitive reserve by performance groups. The explained variance is the total cumulative variance explained by all the predictors in the model. The numbers inside the cells in the “Predictors” area show the importance of each variable in predicting the outcome variable, where the higher the value the higher the importance. The importance is calculated as the relative error in the prediction when a given predictor is excluded from the model. Blank cells denote that these variables were not important in the model. Total variables: the total number of variables that are important to predicting phonemic fluency. CR: cognitive reserve; YA: younger age; OA: older age; LowPF: low phonemic fluency performance; HighPF: high phonemic fluency performance; BNT: Boston Naming Test (spontaneous responses); PCV: PC-Vienna System; PASAT: Paced Auditory Serial Addition Test; TMT A: Trial Making Test A; CTT: Color Trails Test; FRT: Facial Recognition Test; JLOT: Judgment of Line Orientation Test; LM: Logical Memory; VR: Visual Reproduction Test; Luria’s HAM: Luria’s Premotor Functions; Hand Alternative Movements; PF: phonemic fluency.

**Figure 3:**
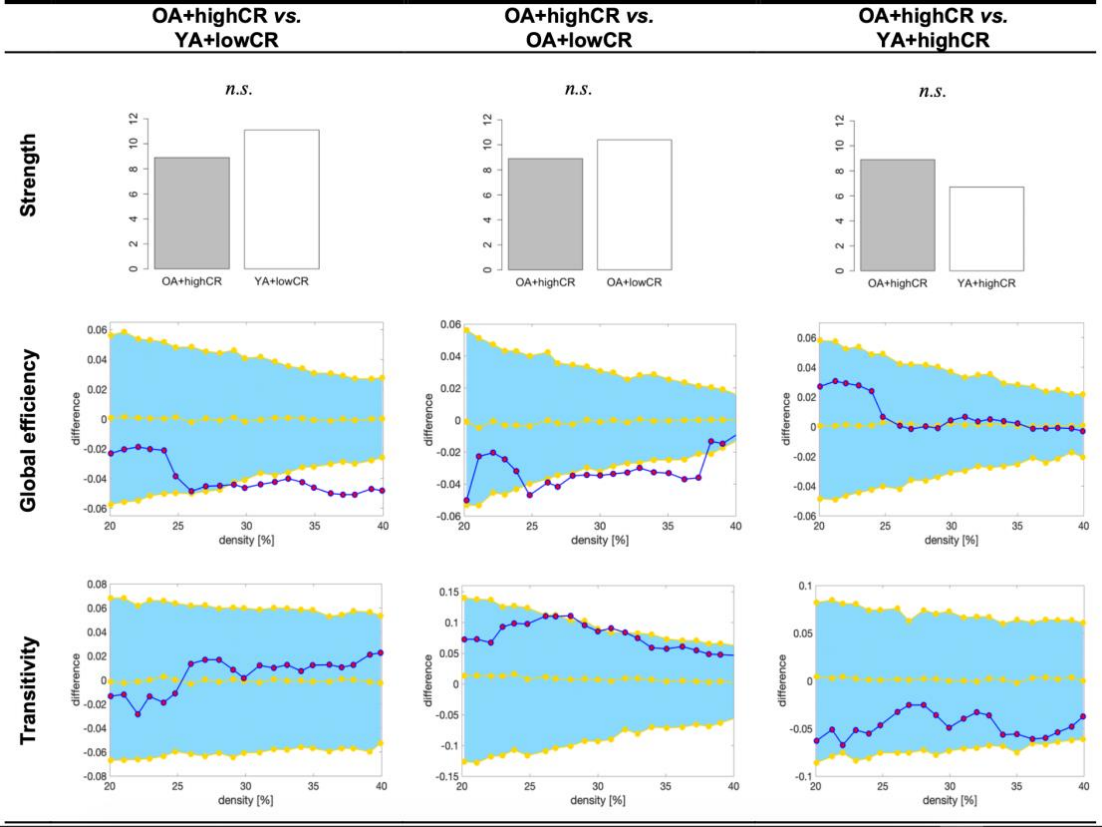
**Graph results for CR by age groups.** For global efficiency and transitivity measures, network densities are displayed on the x-axis from min = 20% to max = 40%, in steps of 1%. Between-group differences in the efficiency measures are displayed on the y-axis. Between-group differences are significant when the red circles fall out of the blue-shaded area. CR, cognitive reserve. HP, high performance. OA+highCR, older age participants with high CR. YA+lowCR, younger age participants with low CR. OA+lowCR, older age participants with low CR. YA+highCR, younger age participants with high CR. *n.s.*, non significant results (p>0.05).

In order to reduce the number of comparisons as part of our third aim, following the finding from the random forest models above, the OA+highCR group was compared against the OA+lowCR and YA+lowCR groups, across the graph measures. Because our interest was to understand why individuals achieve higher performance, all these comparisons were restricted to high performance groups. All these analyses were controlled for the effect of sex. There were no significant differences in the average strength of the OA+highCR group as compared to the OA+lowCR and YA+lowCR groups (*p*>0.05) (Figure 3). Global efficiency was increased in the OA+highCR group as compared to both OA+lowCR and YA+lowCR groups. There were no significant differences in transitivity (*p*>0.05).

### The effect of high cognitive reserve is amplified in high-performance individuals, independently of their age

The significant interaction between CR and performance group revealed that the difference between low phonemic fluency (lowPF) and high phonemic fluency (highPF) performance groups was greater in the highCR group than in the lowCR group (Figure 1B). Hence, higher CR increases performance on phonemic fluency, irrespectively of the age (the partial effect of age was controlled for in the ANCOVA).

To achieve our second aim, four random forest regression models were performed separately within each group (lowPF+lowCR, highPF+lowCR, lowPF+highCR, and highPF+highCR) (Table 2B). In the highPF+highCR group, the model explained 13% of the variance and 13 variables contributed to performance in phonemic fluency (Table 2B). The most important variables in predicting performance were Stroop (Inhibition) and Visual reproduction (Immediate). In the lowPF+highCR group, the model explained 17% of the variance and 19 variables contributed to performance. The most important variables in predicting performance were Visual reproduction (False positives) and JLOT. In the highPF+lowCR group, the model explained 45% of the variance and 32 variables contributed to performance. The most important variables in predicting performance were Stroop (Words) and Luria’s motor coordination. In the lowPF+lowCR group, the model explained 50% of the variance and 28 variables contributed to performance. The most important variables in predicting performance were Stroop (Colors) and CTT-Part 1. Hence, highCR groups need a lower number of contributing variables in order to achieve high performance, and the strength of this contribution is the lowest in highPF+highCR individuals (as reflected by the % of variance). When entering sex as an extra predictor, these results were virtually the same, demonstrating that sex does not have any confounding effect in these models.

**Figure 4:**
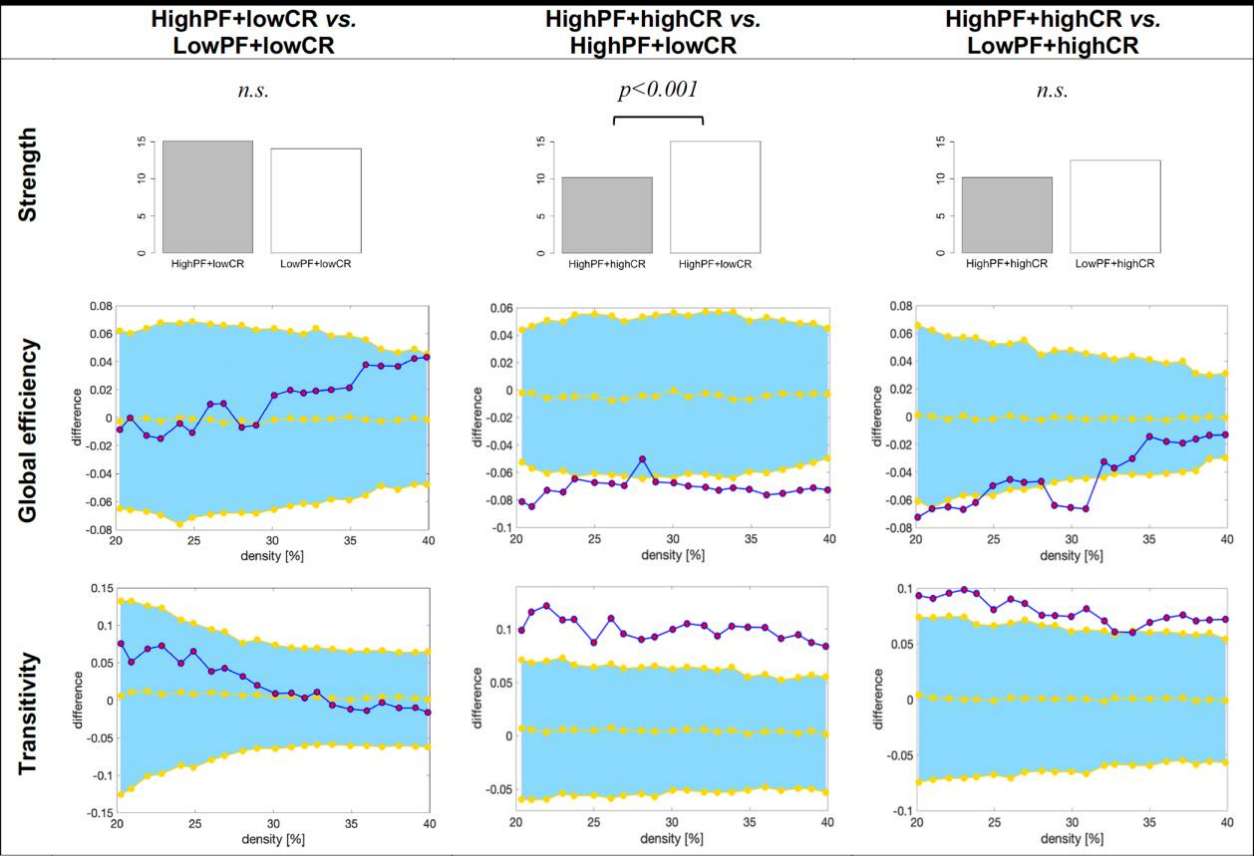
**Graph results for CR by performance groups.** For global efficiency and transitivity measures, network densities are displayed on the x-axis from min = 20% to max = 40%, in steps of 1%. Between-group differences in the efficiency measures are displayed on the y-axis. Between-group differences are significant when the red circles fall out of the blue-shaded area. CR, cognitive reserve. HighPF+lowCR, high performance participants with low CR. LowPF+lowCR, low performance participants with low CR. HighPF+highCR, high performance participants with high CR. LowPF+highCR, low performance participants with high CR. *n.s.*, non significant results (p>0.05).

As in the previous section, we reduced the number of comparisons as part of our third aim by performing follow-up analyses guided by the findings from the random forest models above. We were interested in comparing the highPF+highCR group against the lowPF+highCR and highPF+lowCR groups, as well as in comparing the highPF+lowCR group against the lowPF+lowCR group, across graph measures (Figure 4). All these analyses were controlled for the effect of sex. The highPF+highCR group showed lower average strength than the highPF+lowCR (*p*<0.001), but comparable average strength than the lowPF+highCR group (*p*=0.246). Global efficiency was increased in highPF+highCR as compared with the highPF+lowCR group, and tended to be increased when compared to the lowPF+highCR group. Transitivity was decreased in the highPF+highCR group as compared with both highPF+lowCR and lowPF+highCR groups. When comparing the highPF+lowCR and lowPF+lowCR groups, we did not observe any significant difference in the average strength, global efficiency, or transitivity.

## DISCUSSION

The overall goal of this study was to investigate how CR and efficiency levels contribute to phonemic fluency differently in people with high versus low performance and in younger versus older individuals. We found that older adults performed worse than younger adults in verbal fluency, but this difference was minimized by high CR levels and high efficiency of cognitive networks.

### High cognitive reserve reduces age-related differences in phonemic fluency

Older participants produced fewer words than younger participants, a finding that has repeatedly been reported in previous studies [35, 36]. This reduction in words with increasing age was buffered by high CR levels. High CR levels have been associated with higher performance in phonemic fluency [21–25]. In the current study, we demonstrate that high CR levels minimize the differences in phonemic fluency between younger and older individuals. Indeed, CR is commonly considered as a factor that contributes to maintaining cognitive performance in the presence of increasing age or pathology [7]. We conducted several random forest and graph theory analyses to further understand some of the mechanisms underlying this finding.

The random forest analyses showed that the contribution of various cognitive functions to performance in phonemic fluency differed depending on CR levels and the age. Despite the number of variables contributing to performance was largely the same in high and low CR groups, the strength of this contribution was clearly the greatest in older individuals with high CR levels. Processing speed contributed to better performance in all the four groups. Executive functions substantially increased performance and it was an important contributor to performance in all groups except for older adults with low CR. Interestingly, lexical access contributed to performance only in older adults with high CR levels. The contribution of processing speed, executive functions, and lexical access to phonemic fluency, and better executive functions in individuals with higher CR has been shown in previous studies [12, 14, 16, 18, 19]. The novelty of our study is the signature contribution to verbal fluency associated with CR and age, i.e. lexical access and a strong contribution of executive functions allow for older individuals with high CR to maintain their high performance on verbal fluency.

The graph theory analyses showed that the average global efficiency was increased in older participants with high CR levels. Previous studies have reported higher average global efficiency in individuals with high CR, using graph analysis on neuroimaging data [37, 38]. The novelty in our study is that we report data on the effect of CR on global efficiency stratifying by age, using graph analysis on cognitive data. When calculated on cognitive data, the global efficiency reflects whether cognitive variables correlate with each other in short paths, with higher average global efficiency values reflecting the capacity to quickly distribute information via short paths [39]. In the context of our study, high average global efficiency reflects how the performance in non-fluency tasks contributes to performance in phonemic fluency. Since high CR levels allowed for older individuals to perform better, it is possible that high CR enabled them to rapidly access the lexical storage to retrieve more words (BNT had a high contribution in these individuals), perhaps supported by better executive capacities such as using better strategies, inhibiting distractions, etc. (executive functions also had a high contribution in these individuals). On the contrary, our graph analyses did not show any differences in the average strength or transitivity measures when analyzing CR by age groups. In cognitive networks, the average strength represents the overall magnitude of the correlations among the cognitive measures included in the network. The transitivity reflects how well the nodes are connected to nearby nodes forming cliques, that is, whether our cognitive data tend to be organized into communities of cognitive measures that are strongly correlated to nearby cognitive measures, but weakly correlated to cognitive measures belonging to other communities. Hence, our findings suggest that CR and age groups differ in integration features (global efficiency), rather than in segregation features (transitivity) or the magnitude of the associations among cognitive functions (average strength). Altogether, our findings show that despite largely the same number of cognitive functions contributing to fluency performance in older individuals with high CR levels, they predict a much higher variance of verbal fluency as compared to the other groups. This finding emerges in the absence of significant differences in the average strength or segregation of cognitive networks. It is possible that the healthy nature of our cohort highlights the role of integration features in cognitive compensation, rather than segregation features, which are likely to be related to the reorganization of brain networks seen in neurodegenerative diseases as a consequence of more overt brain pathology [31, 32].

### The effect of high cognitive reserve is amplified in high-performance individuals, independently of their age

An interesting finding of our study is that although individuals with high CR levels performed better, we observed variability with some individuals achieving very high performance and some achieving lower performance. Again, we conducted several random forest and graph theory analyses to further understand the mechanisms underlying this finding.

The random forest analyses showed that individuals with high CR levels need a lower number of contributing variables in order to achieve high performance. Among these, individuals with high CR who achieved lower performance needed a greater number of contributing variables, which contributed to predicting a higher variance of verbal fluency. This finding may suggest that fluency performance partly relies on the number of contributing variables but, also, on the efficiency of the cognitive networks (a lower number of contributing variables and lower predicted variance would suggest more efficient cognitive networks). This is supported by the graph analysis showing that individuals with high CR but low performance had less efficient networks as reflected by higher transitivity values, i.e., a more fragmented cognitive network. Individuals with low CR levels also relied more on processing speed, independently of their age, which we saw in the previous section that it is not the most efficient contribution to verbal fluency. Interestingly, individuals with high CR levels recruited networks involved in visual abilities (immediate visual memory and JLOT). The difference between high CR individuals who achieved very high performance and those who achieved lower performance is that the former recruited executive functions, as already discussed in the previous section, and is also supported by the analyses discussed in this section. These findings may suggest the recruitment of right fronto-parietal networks, which are contralateral to the language networks of the left hemisphere.

Again, these results highlight the lower efficiency of cognitive networks of individuals performing worse, amplified by lower CR levels. The graph theory analyses showed that the signature feature of high CR levels is the lower average strength, and the signature feature of individuals performing better is the less segregated (or fragmented) cognitive networks (lower transitivity). We interpret the finding on lower average strength as a highly efficient network in high CR individuals who are able to achieve high performance by involving the right fronto-parietal network and integrating information in a very efficient manner. In contrast, low CR individuals are much less efficient and their verbal fluency strongly relies on processing speed.

In our previous study, we showed that the contribution of cognitive functions to verbal fluency differed across age groups, and we suggested that this could be due to compensatory processes [9]. In the current study, we confirm that hypothesis and show that high CR and efficiency levels could be at the base of compensatory mechanisms to maintain performance in phonemic fluency with increasing age. Compensation refers to the maintenance or enhancement of performance by recruiting brain areas or networks not normally used for a specific task, as a response to brain deterioration [7] or high cognitive demands [8]. Our findings suggest that older individuals with higher CR levels may have been able to compensate for the negative effect of aging by recruiting brain networks underlying lexical access and using executive networks in a more efficient way. The greater contribution of executive functions in older individuals with high CR levels is supported by the “scaffolding theory of aging and cognition” (STAC) [40]. The STAC theory suggests increased frontal activation with age as a compensatory response. The possible involvement of the right fronto-parietal network discussed above suggests a greater participation of the right hemisphere with increasing age, as postulated by the “hemispheric asymmetry reduction in older adults” (HAROLD) model [41]. The HAROLD model suggests the recruitment of contralateral brain areas as a compensatory mechanism [42, 43]. Recruitment of right fronto-parietal and lexical access networks are also supported by the “Compensation-Related Utilization of Neural Circuits Hypothesis” (CRUNCH), which postulates that new brain regions are recruited, leading to the functional reorganization of the brain.

**Figure 5:**
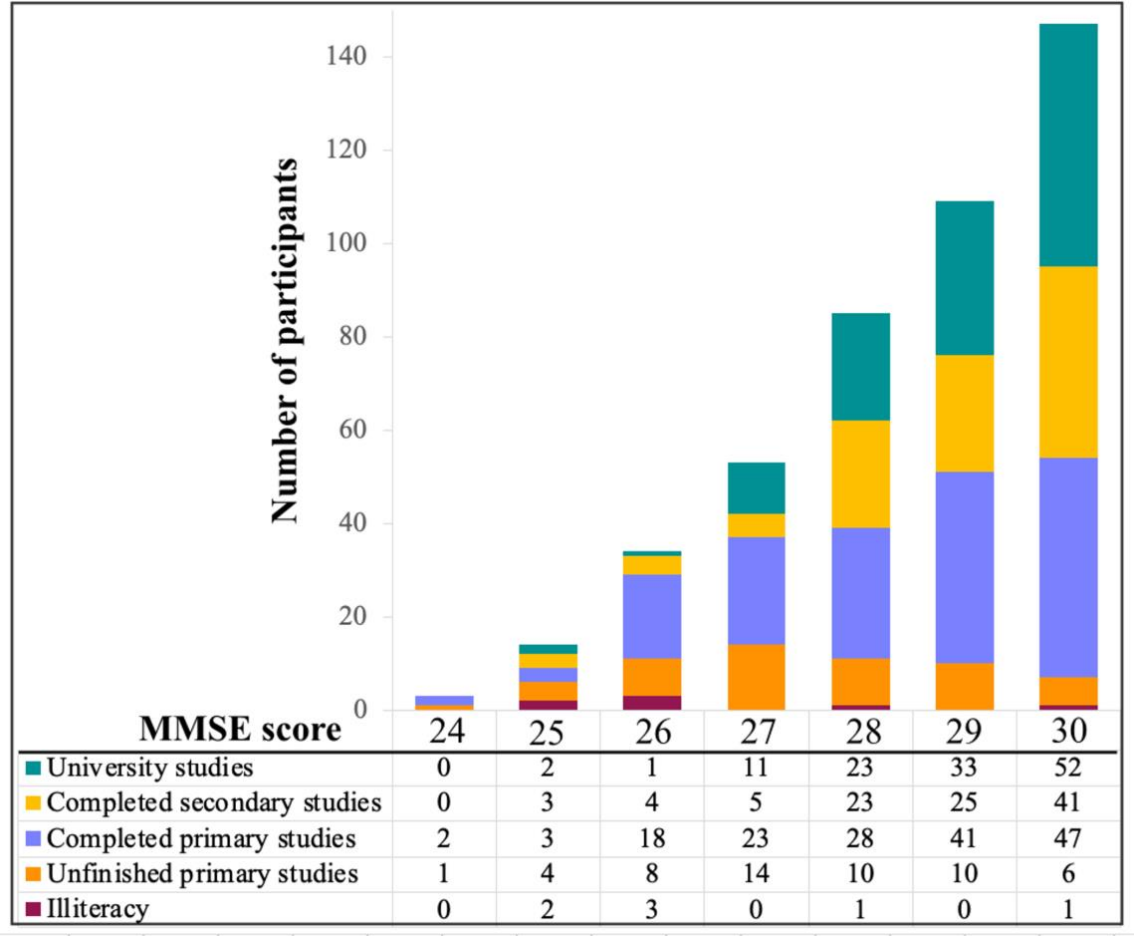
**MMSE scores by education level. MMSE: Mini-Mental State Examination.**

This study has some limitations. We analyzed cross-sectional data. Therefore, our age-related differences may partially be explained by cohort effects. Nonetheless, multivariate analysis methods such as random forest maximize the covariance between the predictors and the outcome variable, being less vulnerable to confounders such as cohort effects [5]. Also, we are currently collecting follow-up data so that our cross-sectional findings can be substantiated in a longitudinal design in our future studies. The literature on graph theory analysis on cognitive data is very limited, and our current study is one of the few published so far. We demonstrate that graph theory shows great potential to deepen the previous cognitive findings obtained using univariate and other multivariate methods. Another consideration is that performance in verbal fluency varies according to the type of stimulus [44]. We used the F-A-S version of phonemic fluency, and our current findings should be replicated using other stimulus such as the P-M-R version, which is also common and validated in the Spanish language [45]. Further, there is currently an ongoing discussion on whether cognitive reserve and compensation occur through a universal brain network or their effects are task-dependent [46]. Our studies are approaching this question by investigating the language function, because language is one of the few functions that can resist the onslaught of aging [1, 2], hence, possibly reflecting the result of successful compensatory mechanisms. While comprehension, semantic abilities, and vocabulary remain rather stable or even improve with age [2, 4], verbal fluency and naming decline with age [5]. We have repeatedly seen in our cohort that naming is the language component most vulnerable to age [5, 47–49]. Therefore, we focused on verbal fluency, which also provides the opportunity to compare different fluency modalities. In our previous study, we demonstrated that phonemic fluency, semantic fluency, and action fluency have different age-dependent trajectories [9]. In particular, performance in semantic fluency and action fluency showed a prominent decline with age, while phonemic fluency showed some decline with age but also showed signs of stability [9]. These characteristics make phonemic fluency an ideal cognitive function to investigate compensatory processes. However, future studies should extend our current analyses to other language components such as naming, as well as to other non-language cognitive functions. Applying random forest and graph theory analyses to different cognitive functions in the future will help to substantiate our current findings, contributing to answer the question on a universal network *vs.* task-dependent networks underpinning cognitive reserve and compensation. Also, extending our cognitive network analyses to neuroimaging measures is warranted in the future in order to better understand the neural correlates of our current findings. We used group-level analysis in graph measures (low *vs.* high fluency performance). This is the most common form of studying network topology. However, future work should explore methods that can generate individual networks [50], enabling correlations between network measures and performance in verbal fluency, age, and CR as a continuous variable. We used the WAIS-III Information subtest and our findings should be tested using other proxies of cognitive reserve. A final consideration is that we excluded individuals with mild cognitive impairment (MCI) using a comprehensive neuropsychological protocol and appropriate normative data. However, we showed that some individuals had MMSE scores in the range 24-26, mostly related to low education. These data can be seen in Figure 5. Including these individuals increases the generalization of our findings to the whole range of education, also including the strata with lowest education. Nonetheless, we acknowledge that other studies using samples with higher education have excluded individuals with an MMSE score below 27 [51].

In conclusion, the current study provides the data to unveil some of the cognitive mechanisms underlying cognitive compensation of verbal fluency during aging. Phonemic fluency decreases less with age in those individuals who have higher CR levels. Our data suggest that the factors determining this finding may include greater capacity to recruit contralateral fronto-parietal networks, and efficiently use ipsilateral language networks, integrating information in a rapid way across less fragmented networks. In terms of functions, these networks are represented by executive/visual abilities and access to the lexicon, respectively. All these abilities can be trained, and CR levels (performance in WAIS-III Information) can also be increased through reading, writing, and learning new materials throughout the lifespan [52]. Hence, this study shows some possibilities for cognitive stimulation of healthy individuals and possibly, also individuals with cognitive impairment. Further, our current results may help to improve clinical interpretation of performance in verbal fluency, as well as serve as an example for future studies on other cognitive functions.

## MATERIALS AND METHODS

### Participants

A total of 446 participants were selected from the GENIC-database (Group of Neuropsychological Studies of the Canary Islands) [5], with ages between 32 and 84 years, and a balanced distribution of sex across age (54.9% females). All participants were assessed with a comprehensive neuropsychological protocol, applied by an experienced neuropsychologist. Afterwards, for each participant, cognitive profile and diagnosis were established at consensus by at least two qualified clinical neuropsychologists, using pertinent age-, sex-, and education-adjusted normative data. The diagnostic procedure consisted on a two-step process: Firstly, we excluded individuals with dementia based on the Blessed Dementia Scale (BDRS [53]) cut point of ≥4, the Functional Activity Questionnaire (FAQ, [54]) cut point of >5, and the Mini-Mental State Examination (MMSE, [55]) score cut point of <24. Secondly, for the specific purposes of this study, we further excluded individuals with MCI based on Winblad’s et al. criteria [56], as applied on our comprehensive neuropsychological protocol. Inclusion criteria for the current study were: (1) normal cognitive performance in comprehensive neuropsychological assessment (2) no neurologic, psychiatric or systemic diseases; and (3) no history of substance abuse. An exception was made for the BDRS. Although the BDRS scale cut-off for abnormality is frequently established at ≥4 points [53, 57], the ‘changes in personality, interests and drive’ subscale may influence the BDRS total score and does not necessarily reflect functional impairment. With the aim of excluding only individuals with functional impairment, we included those participants with total BDRS scores ≥4 (n=24) if: a) 70% or higher percentage of the BDRS total score resulted from the ‘changes in personality, interests and drive’ subscale; and b) if a score ≤1.5 was obtained in the other two subscales (‘changes in performance of everyday activities’ and ‘changes in habits’). The same procedure has been used in previous studies [5, 9, 58]. Hence, all the individuals in this study are cognitively normal. The current study was approved by the ethics committee of the University of La Laguna (Spain), and all participants gave their written informed consent.

### Neuropsychological assessment and cognitive reserve

The neuropsychological protocol includes tests of language, processing speed, attention, executive functions, verbal and visual episodic memory, procedural memory, and visuoconstructive, visuoperceptive and visuospatial functions (Table 3). Among all these tests, the test of phonemic verbal fluency is of special relevance to the current study. Phonemic verbal fluency was assessed with the Controlled Oral Word Association Test (COWAT) [59]. Participants had to recall words that begin with the letters F, A, and S, taking one minute on each of the letters. Proper nouns, numbers, and derived words were scored as intrusion errors. A total score (F+A+S) was calculated as the number of correct words produced, excluding intrusions and perseverations (repetitions of correct words). The other neuropsychological tests and cognitive variables used in this study are listed in Table 3.

**Table 3:** List of predictors (random forest) / nodes (graph analysis), neuropsychological tests, and cognitive components.

**Predictors/Nodes**	**Neuropsychological test**	**Cognitive component**
BNT	Boston Naming Test (BNT) [65]	Lexical access by visual confrontation
PCV - Decision time*	Choice Reaction Time – Motor and Reaction times (PC-Vienna System) [66]	Cognitive and motor reaction times
PCV - Motor time*		
PASAT*	Paced Auditory Serial Addition Test (PASAT) [67]	Maintenance of attention
STROOP Words	Stroop Test [68]	Sheet 1 Words: processing speed
STROOP Colors		Sheet 2 Colors: processing speed
STROOP Inhibition		Sheet 3 Inhibition: executive function
TMT A	Trail Making Test-A (TMT-A) [69]	Focusing/visual tracking
CTT - Part 1	Color Trails Test - Part 1 (CTT-1) [70]	Focusing/visual tracking
CTT - Part 2	Color Trail Test - Part 2 (CTT-2) [70]	Mental flexibility/executive control
FRT	Facial Recognition Test (FRT-brief version) [71]	Visuoperceptive abilities
JLOT - First half	Judgment of Line Orientation Test (JLOT, H form) [71]	Visuospatial abilities
JLOT - Second half		
Digit Span forward	Digit Span – forward and backwards (WMS-III) [72]	Working memory: amplitude
Digit Span backward		Working memory: manipulation
Spatial Span forward	Visuospatial Span – forward and backwards (WMS-III) [72]	Working memory: amplitude
Spatial Span backward		Working memory: manipulation
LM A – Immediate	Logical Memory (LM, WMS-III) [72]	Immediate recall (verbal)
LM B1 - Immediate		Immediate recall (verbal)
LM B2 - Immediate		Immediate recall (verbal)
LM A - Delay		Delayed recall (verbal)
LM B - Delay		Delayed recall (verbal)
LM A - Recognition		Recognition subtests (verbal)
LM B - Recognition		Recognition subtests (verbal)
TAVEC 1^st^ trial	Test de Aprendizaje Verbal España-Complutense (TAVEC, Spanish version of the California Verbal Learning Test (CVLT)) [73]	Immediate recall (verbal)
TAVEC Learning		Immediate recall (verbal)
TAVEC Short delay		delayed recall (verbal)
TAVEC Short delay-Clues		delayed recall (verbal)
TAVEC Long delay		delayed recall (verbal)
TAVEC Long delay-Clues		delayed recall (verbal)
TAVEC Intrusions Delay		
TAVEC Intrusions Delay-Clues		
TAVEC Perseverations*		
TAVEC Recog. Correct		recognition subtests (verbal)
TAVEC Recog. False Positive	
VR I – Total score	Visual Reproduction Test, (VRT, WMS-III) [72]	Immediate recall (visual)
VR II – Total score		Delayed recall (visual)
VR-Copying		2-D visuoconstructive abilities
VR Total Recog.		Recognition subtests (visual)
VR False Positive		
VR Visual discrimination*		Visuoperceptive abilities
Luria’s HAM Right	Luria’s Premotor Functions (Luria’s) [74]	hand alternative movements
Luria’s HAM Left		hand alternative movements
Luria’s - Coordination		motor coordination
Block Design WAIS	Block Design – standard and extended version (WAIS-III) [60]	3-D visuoconstructive abilities

* Nodes excluded from graph analysis. PCV - Decision time and PCV - Motor time were combined as PCV – Total time and included as a single node for graph analysis.

Following previous studies [49, 52], the WAIS-III Information subtest [60], a measure of premorbid IQ, was used as an indicator of cognitive reserve. Among several reserve proxies, WAIS-III Information showed the greatest compensation capacity of the effect of cortical thinning on cognition [52]. Scores in WAIS-III information range from 0 to 28, with higher values reflecting greater capacity.

### Network construction and graph analysis

**Figure 6:**
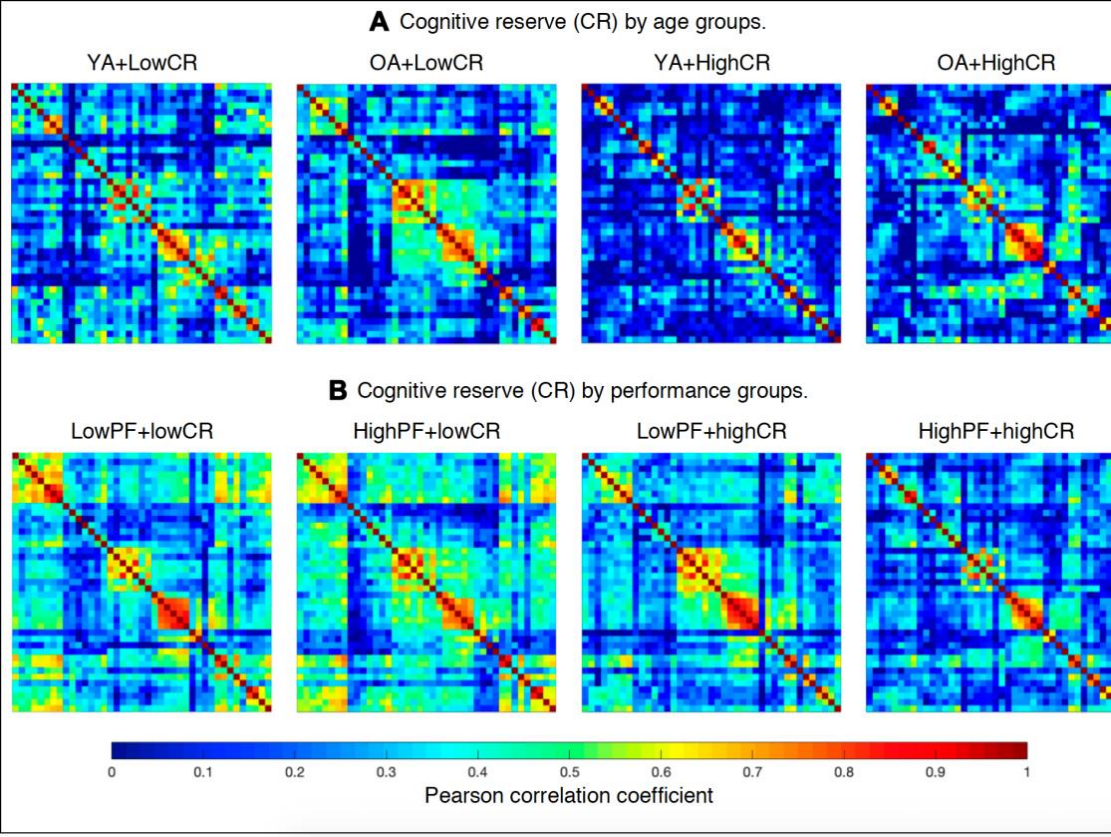
**Weighted correlation matrices (See Supplementary Figures 1–8 for matrices with larger size and labeled regions).** (**A**) Cognitive reserve by age groups: YA+LowCR, younger age group with low CR; OA+LowCR, older age group with low CR; YA+HighCR, younger age group with high CR; OA+HighCR, older age group with high CR. (**B**) Cognitive reserve by performance groups: LowPF+lowCR, low performance group with low CR; HighPF+lowCR, high performance group with low CR; LowPF+highCR, low performance group with high CR; HighPF+highCR, high performance group with high CR. Rows and columns correspond to the correlations between cognitive measures. The color bar indicates the strength of the Pearson correlation coefficients: colder colors represent weaker correlations, while warmer colors represent stronger correlations.

The cognitive variables detailed in Table 3 were selected as nodes for network construction. Performance in these cognitive measures was corrected for the effect of sex using multiple linear regression, and the resulting residual values were used to substitute the raw values for network analysis [61]. As detailed in Table 1, the variables PCV - Decision time and PCV - Motor time were replaced with PCV - Total time as a single node for network analyses. The edges between the nodes were calculated through group-specific association matrices of Pearson correlation coefficients from each pair of nodes (Figure 6, please see Supplementary Figures 1–8 for matrices with larger size and labeled regions). The matrices were binarized by thresholding the correlation coefficients at a range of network densities (min = 20% to max = 40%, in steps of 1%). Both self-connections and negative correlations were excluded. Network topologies were compared across this range, making sure that random topologies and disconnected networks were excluded from the analysis. For this reason, the PASAT, TAVEC Perseverations, and VR Visual discrimination variables listed in Table 3 were excluded, because they were not correlated with the other cognitive variables. Once the networks were constructed, different global measures were calculated: the *average global efficiency* (a measure of integration) and *the transitivity* (a measure of segregation) measures were calculated from the binary networks across the different densities, and the *average strength* was calculated from the weighted network (before binarization). The *average strength* is given by the sum of the weights of all edges connected to a node. In a cognitive network, the average strength represents the overall magnitude of correlations among cognitive measures in the network [28]. The *average global efficiency* is the average inverse shortest path length between a node and the rest of the network, which in contrast to the characteristic path length, can be meaningfully computed on disconnected networks [28]. The average global efficiency measures how efficiently information is exchanged throughout the network [62]. In a cognitive network, the average global efficiency represents whether the performance in non-fluency tasks contributes to performance in phonemic fluency through short paths of correlations. The *transitivity* refers to the fraction of a node’s neighbors that are also neighbors of each other in the whole network, normalized by the whole network. It reflects how well the nodes are connected to nearby nodes forming cliques. In a cognitive network, the transitivity reflects whether our cognitive data tend to be organized into communities of cognitive measures that are strongly correlated to nearby cognitive measures, but weakly correlated to cognitive measures belonging to other communities.

### Statistical analysis

Statistical analyses were performed using the R programming environment [63] and BRAPH (http://braph.org, [64]). We stratified the cohort into groups of CR, performance in phonemic fluency, and age, using the median values of these variables as detailed in Figure 1. We addressed our first aim by testing for the effects of CR level, performance level, and age over phonemic fluency using a factorial analysis of covariance (ANCOVA), including sex as a covariate. We addressed our second aim by using random forest regression analyses to investigate the multivariate association between the measure of phonemic fluency and the 45 cognitive variables detailed in Table 3. In random forest models, the contribution of the predictors in the models is reported as *Imp* (for *Importance*), which reflects the relative error in the prediction when a predictor is excluded from the model. *Imp* values higher than zero denote that a given variable contributes to the prediction of the outcome. The larger the *Imp* value, the greater the contribution. *Imp* values do not have an upper limit and they can rather be interpreted by considering the obtained values in relation to the variable yielding the highest *Imp* value in the model. Our third aim was addressed by comparing the graph measures of average strength, global efficiency, and transitivity across the CR, performance, and age groups.

Two percent of the values was missing across the 45 cognitive variables and were thus imputed. ANCOVA, random forest, and graph analyses were performed on the imputed dataset. For the demographic variables, ANOVA was used for both continuous and dichotomous (dummy) variables and the Chi-square test for categorical variables. P-values in all *post-hoc* analyses were adjusted with the Hochberg’s correction for multiple comparisons. Significant differences were considered when p≤0.05 (two-tailed). Between-group comparisons of graph measures were conducted through 1000 nonparametric permutations over a range of network densities (min = 20% to max = 40%, in steps of 1%). The 95% confidence intervals of each distribution were used as critical values for testing the null hypothesis at p≤0.05 (two-tailed).

## Supplementary Material

Supplementary Figures

Supplementary Table 1
