# Supplementary material for: Cognitive reserve and network efficiency as compensatory mechanisms of the effect of aging on phonemic fluency
Source: Aging (Albany NY). 2020 Nov 17;12(22):23351–78. doi: 10.18632/aging.202177 (PMC7746387; doi:10.18632/aging.202177)
Supplement: Supplementary Figures [file aging-12-202177-s001..pdf]

SUPPLEMENTARY FIGURES

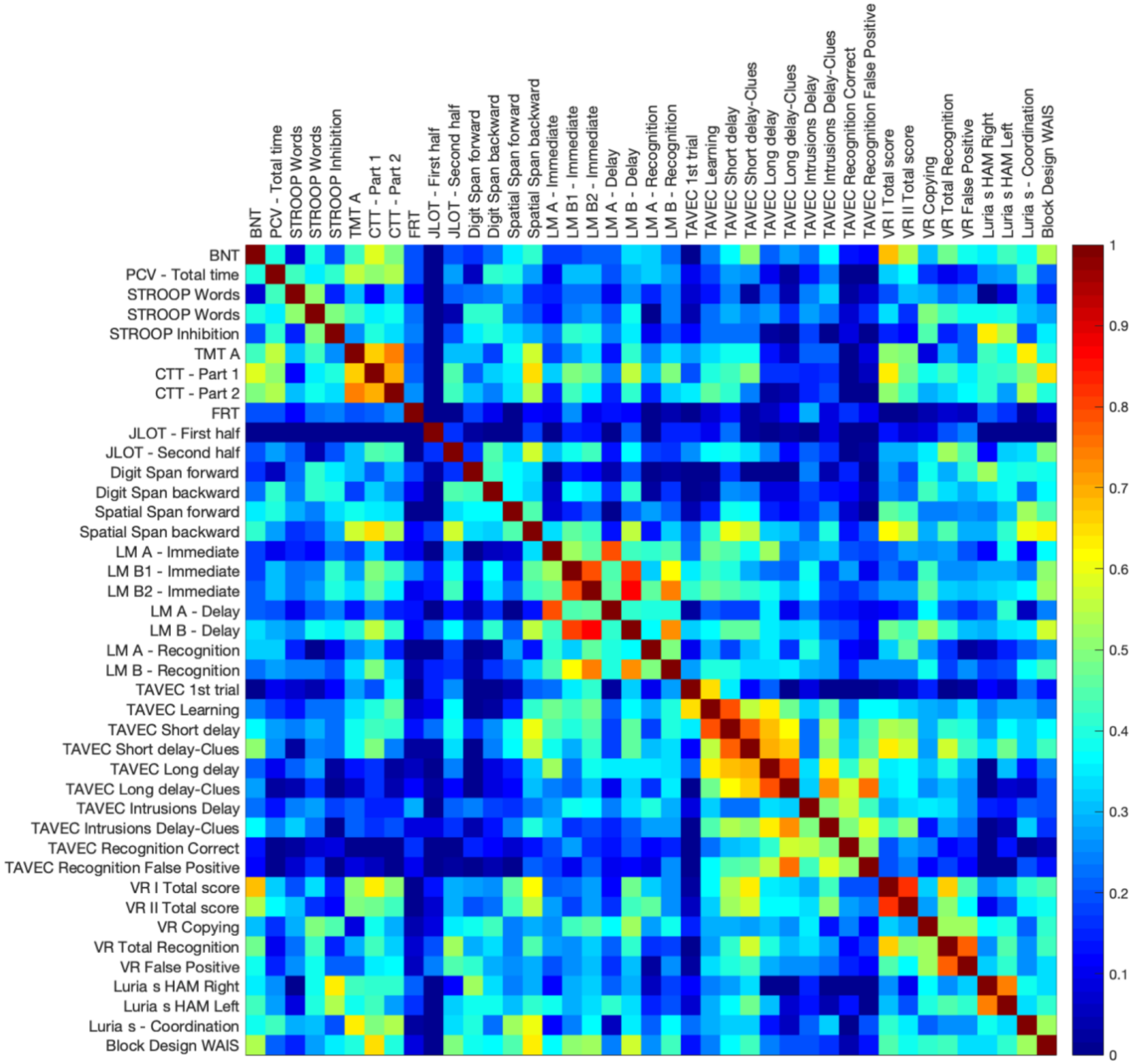

Supplementary Figure 1. Weighted correlation matrix (YA+lowCR). YA, younger age. lowCR, low cognitive reserve. The color bar indicates the strength of the Pearson correlation coefficients: colder colors represent weaker correlations, while warmer colors represent stronger correlations.

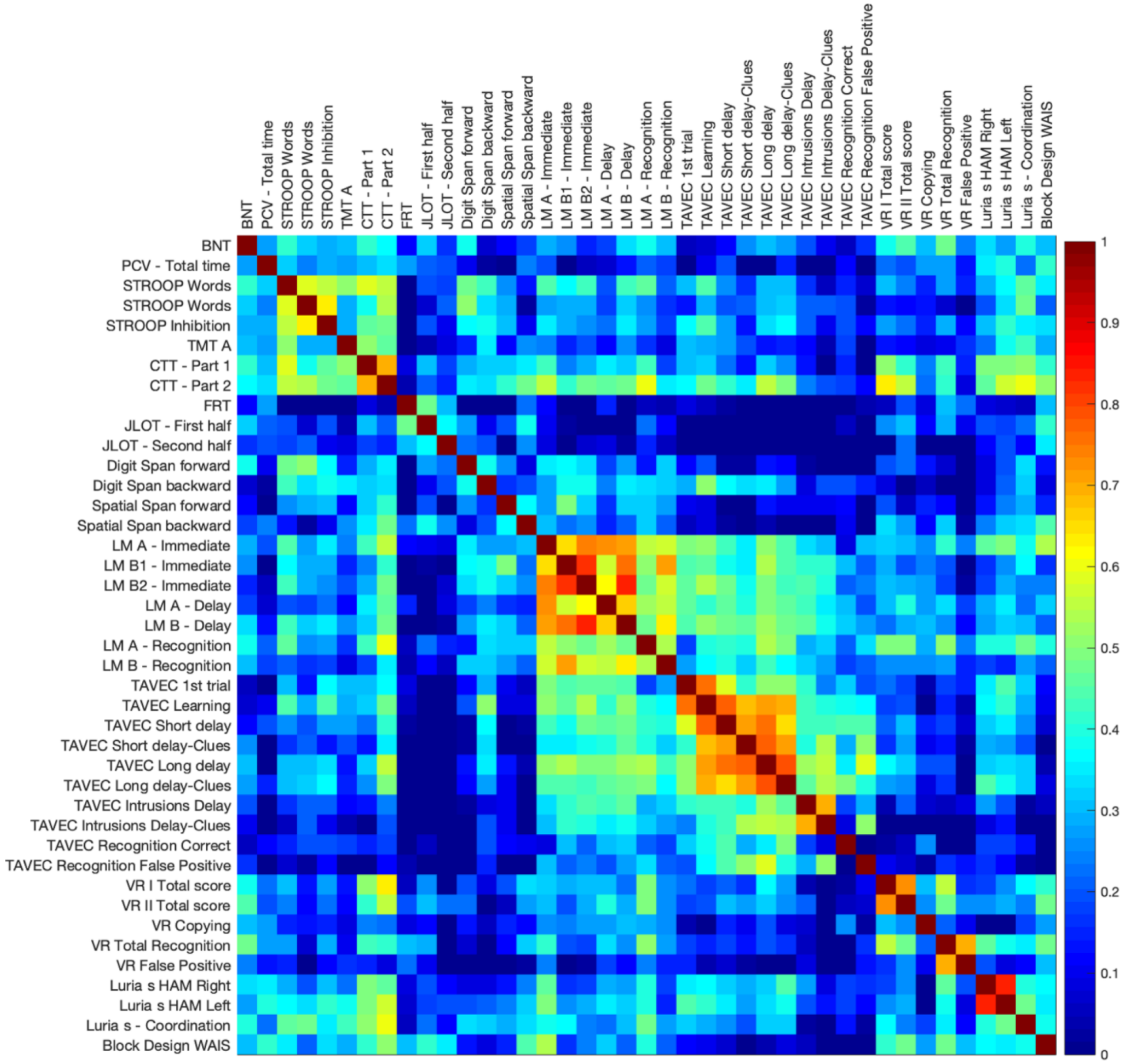

Supplementary Figure 2. Weighted correlation matrix (OA+lowCR). OA, older age. lowCR, low cognitive reserve. The color bar indicates the strength of the Pearson correlation coefficients: colder colors represent weaker correlations, while warmer colors represent stronger correlations.

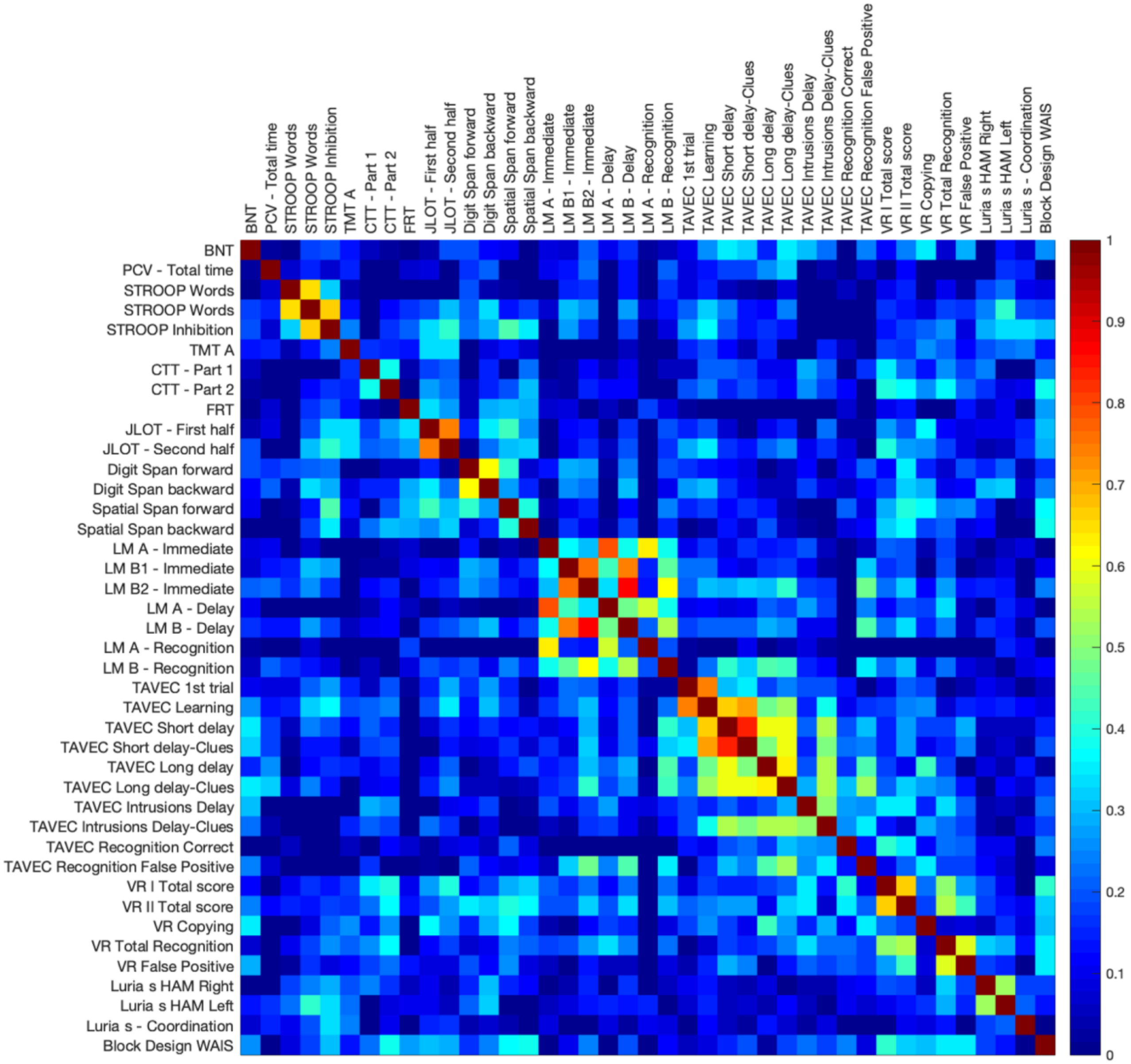

Supplementary Figure 3. Weighted correlation matrix (YA+highCR). YA, younger age. highCR, high cognitive reserve. The color bar indicates the strength of the Pearson correlation coefficients: colder colors represent weaker correlations, while warmer colors represent stronger correlations.

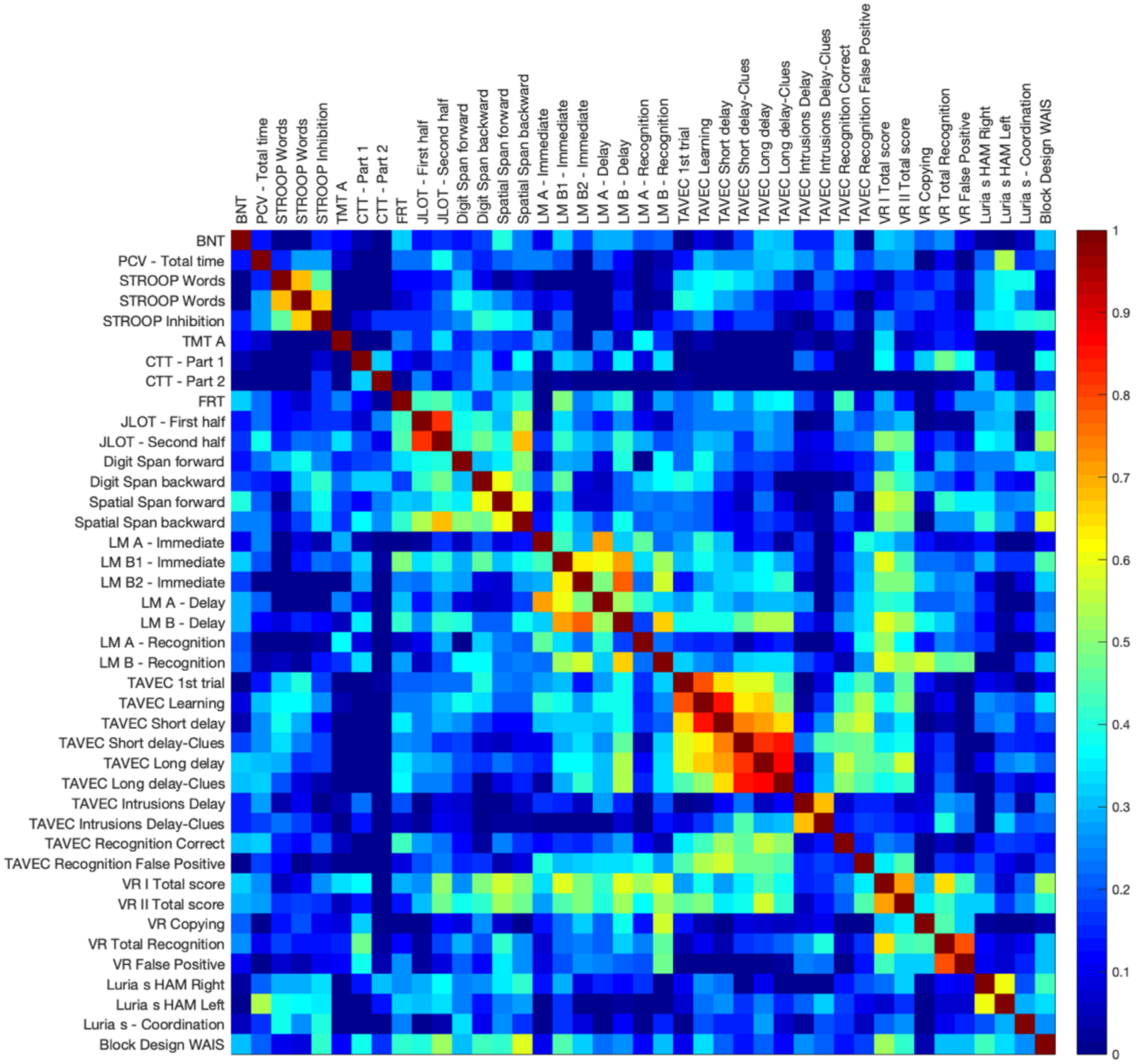

Supplementary Figure 4. Weighted correlation matrix (OA+highCR). OA, older age. highCR, high cognitive reserve. The color bar indicates the strength of the Pearson correlation coefficients: colder colors represent weaker correlations, while warmer colors represent stronger correlations.

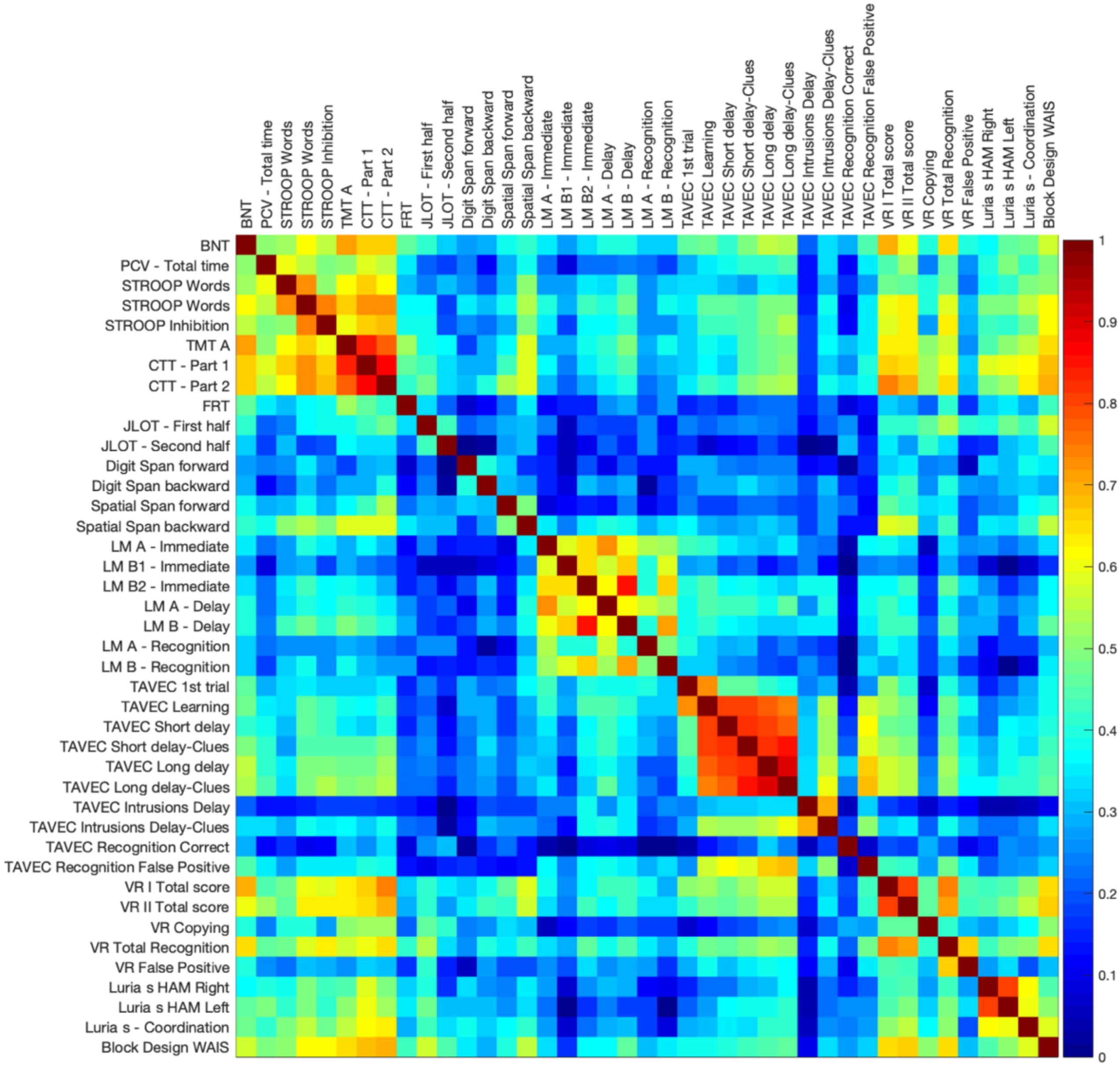

Supplementary Figure 5. Weighted correlation matrix (LowPF+lowCR). LowPF, low phonemic fluency performance. lowCR: low cognitive reserve. The color bar indicates the strength of the Pearson correlation coefficients: colder colors represent weaker correlations, while warmer colors represent stronger correlations.

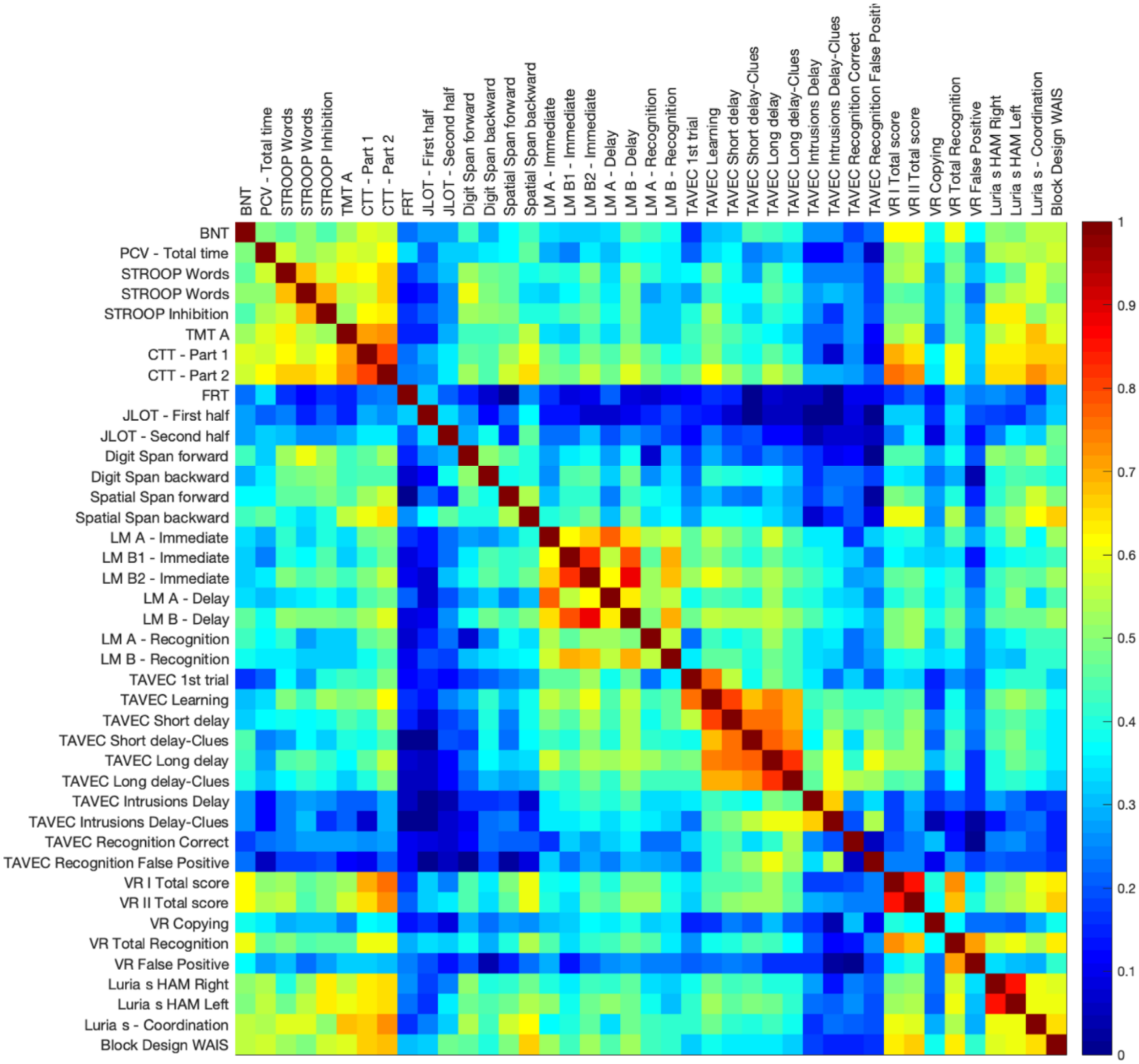

Supplementary Figure 6. Weighted correlation matrix (HighPF+lowCR). HighPF, high phonemic fluency performance. lowCR: low cognitive reserve. The color bar indicates the strength of the Pearson correlation coefficients: colder colors represent weaker correlations, while warmer colors represent stronger correlations.

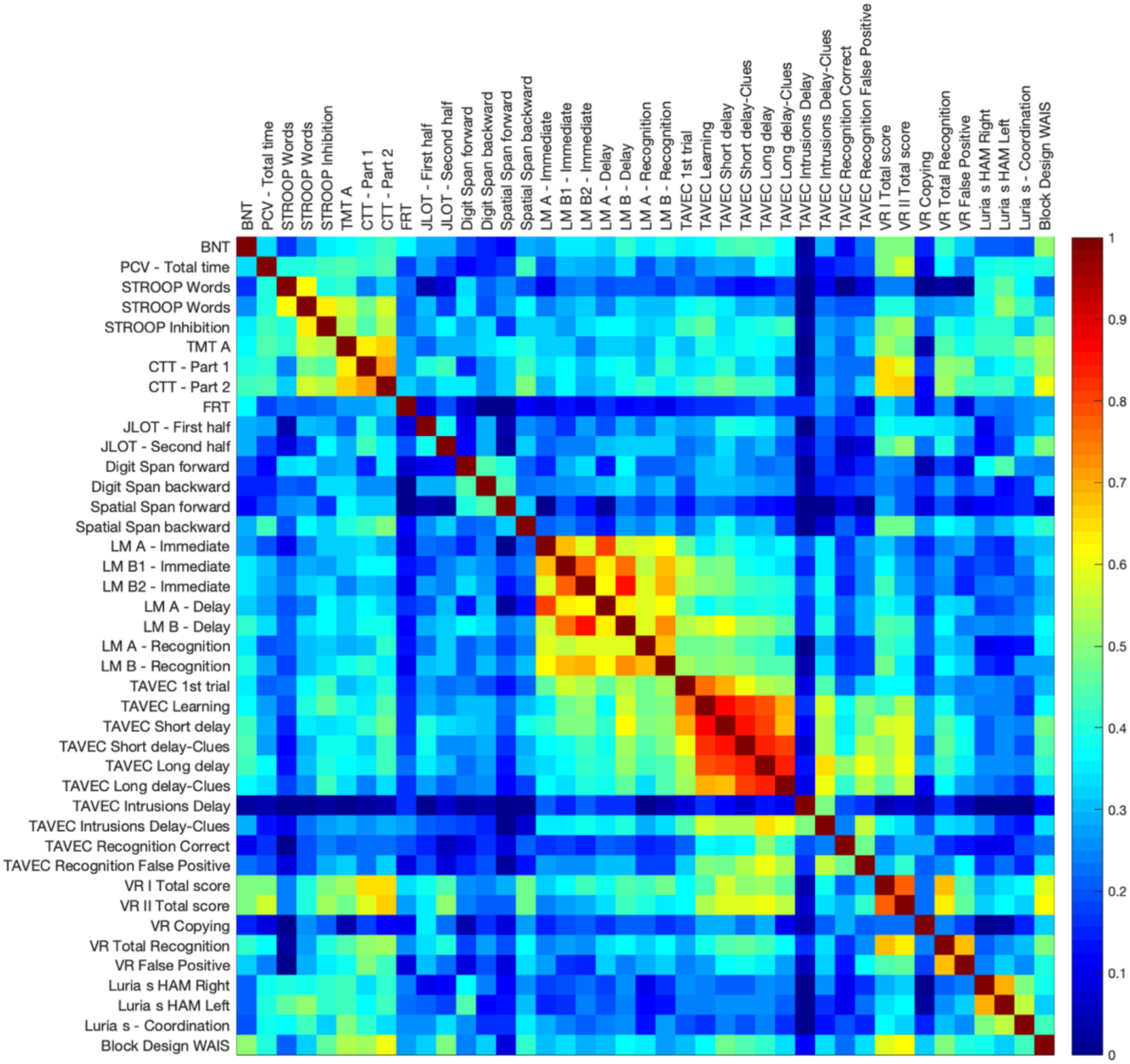

Supplementary Figure 7. Weighted correlation matrix (LowPF+highCR). LowPF, low phonemic fluency performance. highCR: high cognitive reserve. The color bar indicates the strength of the Pearson correlation coefficients: colder colors represent weaker correlations, while warmer colors represent stronger correlations.

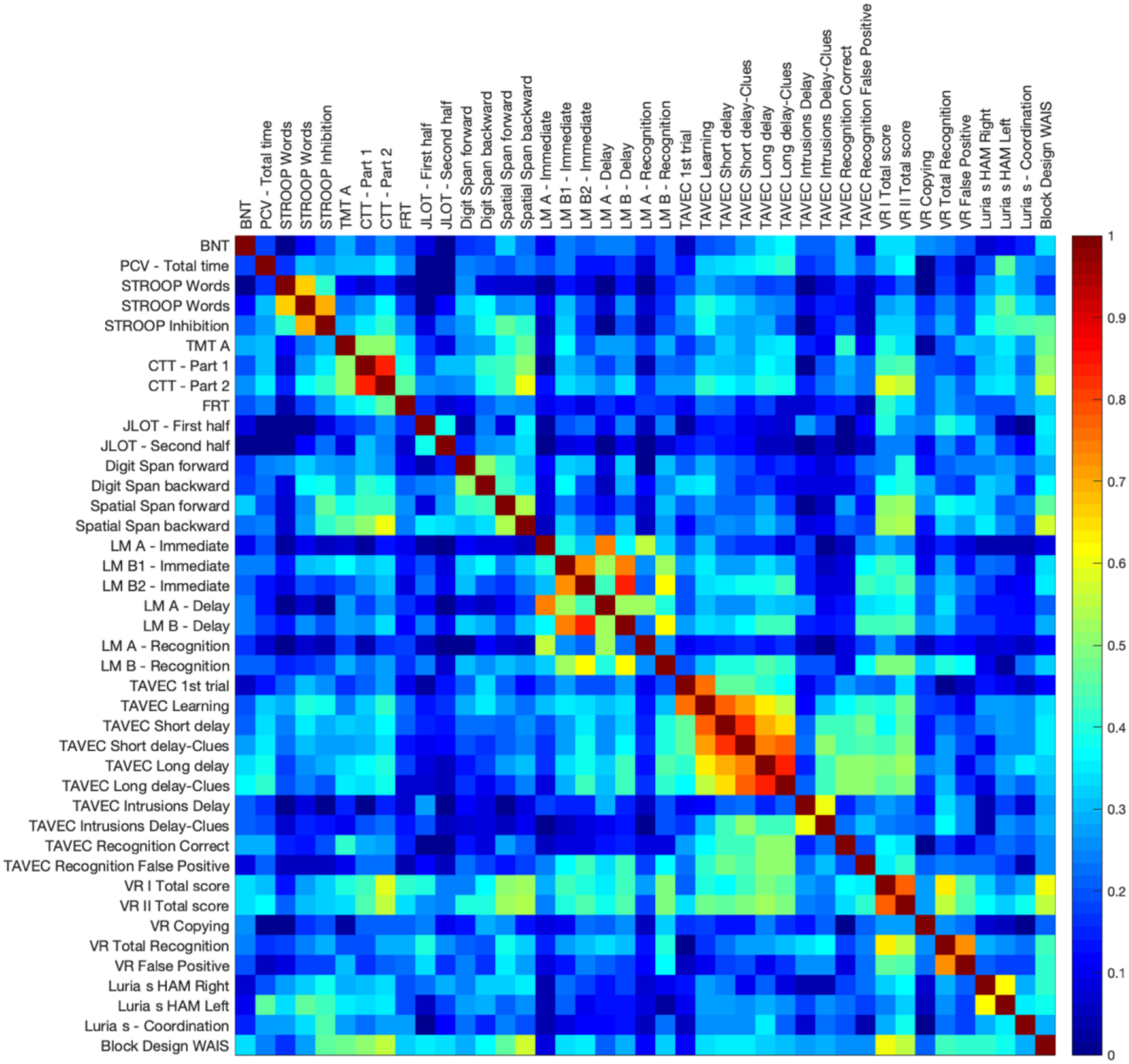

Supplementary Figure 8. Weighted correlation matrix (HighPF+highCR), HighPF, high phonemic fluency performance. highCR: high cognitive reserve. The color bar indicates the strength of the Pearson correlation coefficients: colder colors represent weaker correlations, while warmer colors represent stronger correlations.
