# Supplementary material for: Cognitive reserve and network efficiency as compensatory mechanisms of the effect of aging on phonemic fluency
Source: Aging (Albany NY). 2020 Nov 17;12(22):23351–78. doi: 10.18632/aging.202177 (PMC7746387; doi:10.18632/aging.202177)
Supplement: Supplementary Table 1 [file aging-12-202177-s002..pdf]

SUPPLEMENTARY TABLE

Supplementary Table 1. Performance in neuropsychological test by group.

		Low Cognitive Reserve (lowCR)				High Cognitive Reserve (highCR)			
		Younger-age (YA, n=95)		Older-age (OA, n=129)		Younger-age (YA, n=127)		Older-age (OA, n=95)	
		LowPF	HighPF	LowPF	HighPF	LowPF	HighPF	LowPF	HighPF
	n	50	45	68	61	64	63	50	45
**Neuropsychological test**									
BNT, score	M	24.7	25.5	19.8	21.9	28.3	29.1	26.3	28.0
	SD	3.5	3.2	4.7	3.9	1.7	1.2	2.2	2.5
PCV - Decision time, milliseconds	M	462.4	463.3	576.9	563.1	459.8	456.3	554.5	491.6
	SD	64.3	67.7	121.7	109.9	61.7	61.6	85.9	82.2
PCV - Motor time, milliseconds	M	212.8	191.2	294.1	266.0	191.6	181.3	243.2	225.6
	SD	53.0	72.4	83.1	71.3	54.0	50.9	71.5	62.3
PASAT, score	M	58.7	58.8	58.1	58.4	59.0	59.6	58.6	59.5
	SD	1.5	2.6	3.6	2.1	1.6	1.0	2.2	1.9
STROOP Words, score	M	92.2	103.6	67.7	78.2	103.9	110.7	93.4	106.6
	SD	14.6	18.6	19.9	18.2	14.4	12.8	14.7	14.5
STROOP Color, score	M	64.7	70.9	45.8	53.5	69.1	76.3	57.6	68.7
	SD	10.6	12.7	10.8	14.2	10.5	11.1	11.3	10.6
STROOP Inhibition, score	M	34.8	40.5	22.0	27.6	40.2	44.8	30.8	38.8
	SD	8.4	9.4	7.6	9.9	8.0	8.5	6.5	8.3
TMT A, seconds	M	42.3	36.8	83.9	62.9	33.4	30.0	48.7	41.3
	SD	13.5	12.6	34.6	18.1	8.9	8.3	15.8	16.5
CTT - Part 1, seconds	M	51.9	47.5	105.0	83.8	40.0	36.4	66.0	49.6
	SD	18.4	17.2	34.8	29.1	10.1	10.7	26.0	16.3
CTT - Part 2, seconds	M	117.5	96.0	210.5	172.7	91.3	85.4	135.9	115.7
	SD	46.2	37.2	57.4	50.9	24.7	22.5	42.8	33.4
FRT, score	M	22.2	22.3	20.5	21.4	23.0	23.5	21.7	21.7
	SD	2.1	1.9	2.3	2.0	2.0	2.0	1.9	2.1
JLOT - First half, score	M	12.6	12.9	10.6	11.6	13.9	13.9	13.2	13.4
	SD	2.0	2.3	3.0	2.8	1.2	1.2	1.7	1.4
JLOT - Second half, score	M	8.4	9.4	6.8	8.0	11.2	11.3	9.4	10.3
	SD	2.9	2.7	2.7	2.5	2.4	2.5	2.7	2.7
Digit Span forward, score	M	6.9	8.1	5.8	6.3	8.2	9.5	7.3	8.8
	SD	1.7	1.9	1.3	1.5	1.9	2.2	2.0	2.0
Digit Span backward, score	M	4.6	5.6	3.8	4.2	6.3	7.2	5.4	6.4
	SD	1.4	1.8	1.1	1.4	1.8	2.1	1.9	1.8
Spatial Span forward, score	M	7.3	8.3	6.2	6.6	8.1	8.6	7.5	7.5
	SD	1.6	1.5	1.5	1.7	2.0	1.9	1.7	2.0
Spatial Span backward, score	M	6.6	7.0	4.6	5.2	7.8	8.5	6.6	7.1
	SD	1.5	1.8	1.7	1.5	1.6	1.7	1.8	2.1
LM A – Immediate, score	M	11.0	11.7	8.8	9.7	14.1	14.3	12.1	13.7
	SD	3.0	3.1	3.4	3.8	3.6	3.1	3.9	3.4
LM B1 – Immediate, score	M	8.0	9.5	6.9	7.4	12.4	13.7	9.7	11.6
	SD	3.3	3.9	2.8	3.2	3.6	3.8	3.9	3.2
LM B2 – Immediate, score	M	12.4	14.7	9.9	10.8	16.9	18.4	13.9	16.8
	SD	4.2	4.3	3.3	4.0	3.8	3.6	3.7	3.6
LM A – Delay, score	M	8.4	9.4	5.4	6.1	11.5	12.1	9.0	10.1
	SD	3.8	4.2	3.8	4.0	3.7	4.0	4.2	4.4
LM B – Delay, score	M	11.1	13.2	7.8	9.1	15.7	17.2	12.2	15.8
	SD	4.2	4.3	3.9	4.3	4.0	3.8	3.9	3.6
LM A – Recognition, score	M	11.3	11.4	9.6	10.1	12.4	12.4	11.0	11.6
	SD	2.0	2.2	2.2	2.0	2.0	1.9	2.2	1.9
LM B – Recognition, score	M	12.0	12.7	10.9	11.5	13.9	14.2	12.5	13.4
	SD	2.0	1.6	2.2	1.9	1.3	1.2	2.0	1.4

TAVEC 1st trial, score	M	7.1	6.9	5.5	5.6	7.4	7.5	5.8	7.1
	SD	1.9	1.7	1.8	1.8	1.8	2.0	1.8	2.2
TAVEC Learning, score	M	54.9	56.5	44.8	48.1	58.0	60.2	49.0	54.3
	SD	8.0	7.9	11.2	9.7	8.3	7.8	9.9	10.5
TAVEC Short Delay, score	M	11.7	11.7	8.4	9.8	12.4	13.3	9.8	10.8
	SD	2.6	2.5	3.2	2.9	2.7	2.6	3.3	2.7
TAVEC Short Delay-Clues, score	M	13.0	12.9	9.8	11.0	13.6	14.2	11.4	11.9
	SD	2.2	2.6	3.4	2.6	2.4	2.1	3.1	2.6
TAVEC Long Delay, score	M	13.6	13.7	9.9	11.1	14.4	15.0	11.5	12.7
	SD	2.1	2.2	3.8	3.0	2.0	1.8	3.3	3.2
TAVEC Long Delay-Clues, score	M	14.6	14.4	11.1	12.4	15.0	15.2	12.5	13.4
	SD	1.6	1.9	3.7	2.7	1.5	1.3	3.1	3.1
TAVEC Intrusions Delay, score	M	3.9	3.7	5.3	5.4	3.0	3.5	3.7	4.4
	SD	3.3	2.9	4.8	5.5	3.0	3.1	3.8	3.9
TAVEC Intrusions Delay-Clues, score	M	1.8	1.8	4.4	3.2	0.9	1.1	2.4	1.9
	SD	1.9	2.1	3.7	3.4	1.2	1.2	2.5	1.9
TAVEC Perseverations, score	M	5.3	6.7	6.8	6.3	6.3	3.7	4.9	4.8
	SD	6.2	5.9	6.7	5.7	6.7	3.6	3.7	3.5
TAVEC Recognition Correct answers, score	M	15.5	15.7	14.8	15.0	15.7	15.8	15.2	15.4
	SD	0.7	0.5	2.0	1.6	0.6	0.4	1.1	1.2
TAVEC Recognition False Positives, score	M	1.0	1.0	2.9	1.7	0.5	0.6	1.1	1.1
	SD	0.9	1.0	2.9	1.8	0.7	0.7	1.6	1.0
VR I – Total score	M	78.3	82.0	52.2	55.9	88.4	89.6	70.9	76.7
	SD	12.7	14.1	16.0	18.5	8.6	8.7	15.6	13.9
VR II – Total score	M	62.9	67.0	27.9	31.9	76.0	80.1	45.8	55.8
	SD	19.4	19.5	15.0	18.3	16.4	15.0	19.8	18.6
VR-Copying, score	M	99.2	99.2	93.4	95.8	100.0	100.3	99.0	99.6
	SD	3.6	2.6	8.4	7.0	2.5	3.0	3.4	3.0
VR Total Recognition, score	M	43.7	44.4	38.6	40.1	44.8	45.5	42.7	43.8
	SD	3.1	3.0	3.1	3.3	2.3	2.4	3.1	2.8
VR False Positives, score	M	2.4	2.0	4.3	3.7	1.8	1.3	2.7	2.2
	SD	2.1	1.7	2.7	3.2	1.4	1.1	2.3	1.9
VR Visual discrimination, score	M	6.5	6.4	6.3	6.7	6.7	6.6	6.7	6.7
	SD	0.6	0.7	0.9	0.6	0.5	0.5	0.5	0.6
Luria’s HAM Right, score	M	15.2	17.9	9.9	10.8	17.1	19.4	13.3	15.9
	SD	5.0	5.5	3.6	4.2	5.4	4.9	4.4	6.1
Luria’s HAM Left, score	M	16.6	18.3	10.7	11.7	17.8	20.0	13.9	16.2
	SD	4.4	5.3	4.1	4.4	5.7	4.7	4.9	5.7
Luria’s – Coordination, score	M	47.9	48.7	24.0	27.3	54.9	61.9	41.7	46.6
	SD	17.3	15.9	14.4	12.9	15.6	16.9	17.0	18.4
Block Design WAIS, score	M	32.6	37.3	19.3	22.9	43.3	46.4	30.3	34.7
	SD	8.6	9.5	6.9	9.0	9.9	9.4	8.8	10.4

CR: cognitive reserve; YA: younger age; OA: older age; LowPF: low phonemic fluency performance; HighPF: high phonemic fluency performance. M: Mean; SD: Standard Deviation; BNT: Boston Naming Test (spontaneous responses); PCV: PC-Vienna System; PASAT: Paced Auditory Serial Addition Test; TMT A: Trial Making Test A; CTT: Color Trails Test; FRT: Facial Recognition Test; JLOT: Judgment of Line Orientation Test. LM: Logical Memory; VR: Visual Reproduction Test; Luria’s HAM: Luria’s Premotor Functions; Hand Alternative Movements; PF: phonemic fluency.
